# Ocular complications and associated factors among traditional eye medicines users attending the eye clinic at Mulago national referral hospital, Uganda

**DOI:** 10.4314/ahs.v23i2.53

**Published:** 2023-06

**Authors:** Fransisco Msonge, Lusobya Rebecca Clare, Anne A Musika, Immaculate Atukunda, Caroline Otike, Juma Paul, Elizabeth Nagawa, Eunice Headcraph, Lydia Nakiyingi, Charles M Yancey, Agaba John, Juliet Otiti-Sengeri

**Affiliations:** 1 Department of Ophthalmology, School of Medicine, Makerere University College of Health Sciences, Kampala, Uganda; 2 Clinical Epidemiology Unit, School of Medicine, Makerere University College of Health Sciences, Kampala, Uganda; 3 Department of Medicine, College of Health Sciences, Makerere University, Kampala, Uganda; 4 West Metro Ophthalmology, Golden Valley, Minnesota USA

**Keywords:** Traditional eye medicine, Uganda, Complications

## Abstract

**Background:**

Over the past decades there has been a phenomenal increase in the use of Traditional Eye medicines (TEM) worldwide and there are several factors that compel patients to use TEM.

**Objectives:**

We conducted a study to determine the types of traditional eye medicine, ocular complications, and associated factors among traditional eye medicine users at the Mulago National Referral Hospital (MNRH) eye clinic.

**Methods and Materials:**

A hospital-based cross-sectional study among TEM users at MNRH eye clinic from June to August 2021. Epi Data version 4.2 and STATA version 15 used for analysis. A modified Poisson regression with robust standard errors was used to determine the associated factors.

**Results:**

Overall, 182 TEM users (males:53.3%) were enrolled, with a mean age of 36±21SD years. The most frequently used type of TEM were plant products (47.8%). 70% of TEM users had ocular complications, the most frequent manifestation was conjunctivitis (53.9%). Ocular complications were significantly associated with living in the urban areas (p< 0.006) and participants who reported ease and availability of TEM (p < 0.001).

**Conclusion:**

Plant-based products were the most frequently used types of TEM, a large proportion of the TEM users were found with sight-threatening ocular complications.

## Introduction

There is a growing demand for eye care in developing countries and many individuals turn to Traditional Eye Medicine (TEM) for care and treatment for several reasons 1. TEM is used in sub-Saharan African nations in 33.8% of patients 2. It has been found that 13.2% of patients had used TEM and perhaps only came to the hospital when initial treatment did not yield the desired results 3. The use of TEM is recognized as an important contributing factor for delay in treatment 4, 5.

Different types of TEMs, such as plant, animal, human and chemical have been documented across the globe. The commonly used TEM are liquids from plant leaves and roots and other concoctions of unknown origin 3. These substances may include liquid, human urine, powdery substance, and Holy water 3. In one study, the most frequently used TEMs was kerosene (28.2%) this is similar to what was found in eastern Nigeria 6 were the commonest TEMs used were chemical substances 7 [WU1]. However, in other studies conducted in India and Africa 7 8 breast milk was the commonest TEMs used

These (TEMs[WU2])were more often prescribed by non-traditional practitioners (66.4%) than traditional (36.9%) practitioners 6,9. TEM is associated with ocular complications including corneal opacities, conjunctivitis, anterior staphyloma, and corneal ulcers, endophthalmitis, cataract 2, 4, 9-12 and a report from a teaching hospital in Nigeria showed that complications occurred in 54.8% of the subjects 2. While from Harare, Zimbabwe it was noted that the use of TEM was associated with specific ocular complications in 58.6% of the cases 9.

A report from different works of the literature shows that rural dwellers are more likely to use TEM than urban dwellers. 2, 13 In another study the occupations of the patients were; Farmer, Student, Trader, Civil Servant, Artisan, and Unemployed participants. 7 and the risk factors for TEM use included age above 50 years 3.

Anecdotal reports in Uganda show that Traditional eye medicine use is a significant problem and contributes to the burden of visual loss. However, data on TEM use in Uganda is still scarce and no study has been done at Mulago Hospital. This study aimed to determine the types of traditional eye medicines, ocular complications, and the factors associated with ocular complications among patients attending Mulago National Referral Hospital eye clinic.

## Methods

### Study design and setting

This was a hospital-based, cross-sectional study conducted at the ophthalmic outpatient clinic of Mulago National Referral Hospital (MNRH), Kampala, Uganda. The Hospital is located about 5 km from Kampala city center. MNRH ophthalmic outpatient clinic receives over 1450 outpatients per month and 17,400 in a year and there are several super-specialized clinics.

### Study population

The study included all patients irrespective of age, who used TEM and presented with ocular signs and symptoms at MNRH- ophthalmic outpatient clinic in three months of the study period. We excluded all participants who were too ill to undergo a full ophthalmic examination.

### Data collection procedures and instruments

Data were collected in four working days, by the principal investigator with the help of two trained research assistants. All incoming patients attending the eye clinic were seen by different doctors at the clinic and informed about the study and the purpose of the study and as part of doctor-patient consultation, they were asked about TEM use. Only those who verbally confirmed to have used TEM and then signed informed consent or assent were consecutively enrolled in the study until the sample size was reached.

A structured questionnaire was used to collect information from participants. The data included; Socio-demographic data, history of TEM use, duration of use, details, and description of the types of TEM used, types of TEMs were categorized into three categories: A.Organic substances, including; plant products, human products, and animal products, B. Inorganic substances, including; chemical products. C. Concoctions of unknown origin (mixed substances), source of TEM, clinical and social reasons for using traditional eye medicines

Physical examination and laboratory data; on blood pressure, pulse rate, respiratory rate, blood sugar, and body temperature, were also obtained.

The principal investigator performed an ophthalmic evaluation for all study participants, the following data were collected:

Vision assessment; Visual acuity at distance was tested using a Snellen chart at 6 meters for school-going children and adults (literate) and an E chart for illiterate. Preferential looking tests by using different visual stimuli at the clinic were done to quantitate visual acuity in preverbal infants. Lea test; the grating for 6-24 months and Cardiff test for 18-60 months was used to take visual acuity.

Each eye was occluded in turn and the best visual acuity (VA) was assessed, those found with VA worse than 6/6, the pinhole was used and subjective refraction was done to those who showed improvement on pinhole, so to get the best-corrected visual acuity.

External eye examination was done by penlight torch. Tonometry in children was being done using an ICARE (handheld digital tonometer) and Goldman applanation tonometer in adults.

Anterior segment was examined by using a slit lamp (Zeiss and Haag Streit type present at the clinic) and a portable slit lamp biomicroscope in children. Staining the tear film with fluorescein stain was done to assess ocular surface abnormality.

Biomicroscopic examination of the fundus was done using Volk lenses (+ 90.0D) or indirect ophthalmoscope after dilation of the eye with a cyclopentolate 1% eye drop. The principal investigator ensured that all required examination and documentation were done and completed before letting the participant leave the clinic and appropriate treatment was given to all patients where needed

### Data management and analysis

Collected data were coded and entered into electronic Epi Data version 4.2. The cleaned data were exported to STATA version 15 for analysis. Baseline characteristics were summarized using means with their standard deviations and medians with the 25th and 75th percentiles for numerical variables and proportions for categorical variables, modified Poisson regression with robust standard errors were used for the analysis of factors associated with ocular complications among TEM use. The p < 0.05 was considered statistically significant in, bivariate and multivariate analysis.

## Results

**Figure 1:**
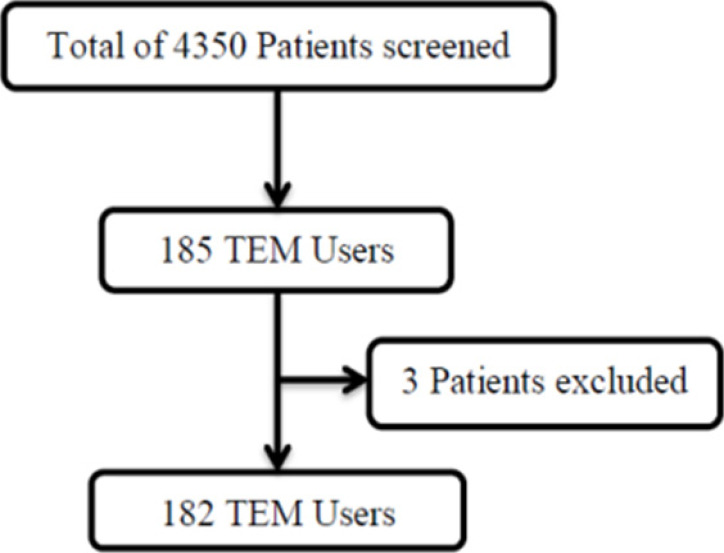
Study participants flow chart

A total of 4350 patients at the eye clinic were assessed for eligibility, of which 185[WU3] reported use of TEM (Figure 1). Three of these were excluded for being too ill to withstand the rigor of the interview and assessment, leaving a total of 182 eligible for the study. Of 182 eligible participants (males were 53.3%), the mean age was 36± 21 SD years and ranged from 0.2 years to 76 years, majority aged between 21 and 40 years 31.9% (58/182). Married were 60.7% (94/155), traders were 29.1% (53/182), and resided in the rural areas 52.2% (95/182). See Table 1

**Table 1:** Social demographic characteristics of the traditional eye medicine users(n=182)

Characteristics	(n)	(%)
**Age(years)**		
0-20	46	25.2
21-40	58	31.9
41-60	51	23.8
61-80	27	14 .8
**Gender**		
Female	85	46.7
Male	97	53 .3
**Residence**		
Rural	95	52.2
Urban	87	47 .8
**Marital status(n=155**[WU4]**)**		
Single	38	24.5
Married	94	60.7
Divorced	7	4.5
Separated	9	5.8
Widowed	7	4.5
**Education status(n=157)**		
None	11	7.0
Primary	82	52.2
Secondary	50	31.9
Tertiary	14	8.9
**Occupation**[WU5]		
Minors	25	13.7
Artisan	15	8.2
Civil servant	11	6.0
Farmer	28	15.4
Traders	53	29.1
Unemployed	25	13.7
Retirees	5	2.8
Others	20	10.9

### Types of TEM

**Figure 2:**
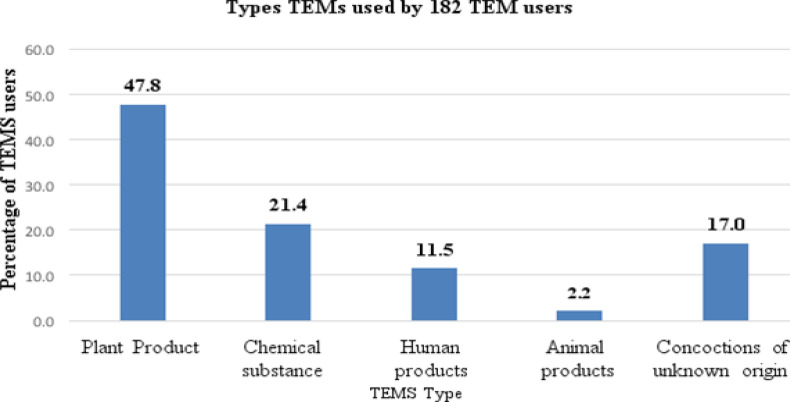
Different types of traditional eye medicine used by 182 traditional eye medicine users

A total of 21 types of TEMs were used by the 182 TEM users, of these 90% (19) were in liquid forms, the remaining 10% (2) were in powdered form. Most of the study participants used plant products (47.8%), followed by chemical substances (21.4%). The commonest plant liquid substances and chemical substances used were Ssere (Bidens pilosa) (n=6) and Salt solution (n=6) respectively. (Figure 2 and Table 2).

**Table 2:** Details of the traditional eye medicine used by study participants

Types TEMs: (Scientific / local names)		
		(%)
	(n)	
**A. ORGANIC SUBSTANCES**		
**Plant liquid**		
**substances**	2	7.4
	4	14.8
Hoslundia opposita (Kamunye leaves)	2	7.4
&xa0;	3	11.1
Momordica foetida (Bombo)	2	7.4
Herbal mixture (Kyogero)	3	11.1
Coffeeleaves (Emwanyi)	1	3.7
Leonotis nepetifolia (Ekifumufumu leaves)	3	11.1
	1	3.7
Plectranthus cyaneus	6	22.2
(Ekiwankulata)		
Bothriocline tomentosa (Ettwatwa)	1	50.0
GynandropsisGynandra (Jjobyo)	1	50.0
Phaseoluslunatus (Kayindiyindi)	3	37.5
Bidenspilosa (Ssere)	6	62.5

**Powdered plant material**		
Plectranthus		
cyaneus (Ekiwankulata)		
Mixed powder		
**Human product**		
Breast milk		
Human urine		
**Animal Product**		
Animal milk	4	100.0
**B. ORGANIC SUBSTANCES**		
**Chemical substance**		
Car engine oil	3	16.7
Cataract clear eye drop.	3	16.7
Halite or rock salt solution (Kisula solution)	3	16.7
Paraffin	3	16.7
Salt solution	6	33.3
**C.CONCOCTIONS OF UNKNOWN ORIGIN**		
Concoctions (mixed substances of unknown origin)	15	17.0

Some participants could not describe the nature of Traditional Eye medicine they used.

The majority of the study participants obtained their TEM from non-traditional healers (64%) and the rest from traditional healers (36%). Of non-traditional healers were mainly from relatives (17.6%), parents (12.1%), and friends (11.5%). Most of the study participants reported having used TEMs on account of tearing 62.6% (114), itching 50.5% (92) followed by photophobia 44.5% (81) [WU6] Ease and availability of TEM 56.6% (103), limited access to health facilities 51.5% (93), and ignorance of the toxic effects 50.5% (92) were the main social reasons for using TEM in our study population.

### Ocular complications

Of 182 TEM users evaluated, complications were found in 70% (127). The commonest ocular manifestation was conjunctivitis 53.9% (98), followed by corneal ulcer 28% (51) and endophthalmitis 5.5% (10). Table 3

**Table 3:** Ocular complications among 182 traditional eye medicine users

Ocular complications	(n)	(%)
Conjunctivitis	98	53.9
Corneal ulcer	51	28.0
Endophthalmitis	10	5.5
Anterior staphyloma	8	4.4
Corneal perforation	6	3.3
Blepharitis	6	3.3
Descemetocele	3	1.7

### Factors associated with ocular complications among TEM users

Participants who reported ease and availability of TEM were 53% more likely to have ocular complications than those who did not (aPR=1.53 95% CI 1.23 - 1.92). Ocular complications were significantly associated with living in the urban areas (p< 0.006) and participants who reported ease and availability of TEM (p < 0.001). Table 4

**Table 4:** Factors associated with ocular complications among 182 traditional eye medicine users

	cPR (95% CI)	p value	aPR (95% CI)	p-value
**Sex**				
Male	1		1	
Female	0.83 (0.68-1.01)	0.067	0.85 (0.71 - 1.03)	0.094
**Residence**				
Rural	1		1	
Urban	1.5 (1.14-1.98)	0.004	**1.46 (1.12 - 1.91)**	**0.006**
**Availability of TEM**				
No	1		1	
Yes	1.52 (1.22-1.89)	<0.001	**1.53 (1.23 - 1.92)**	**<0.001**
**Itching**				
No	1		1	
Yes	0.98 (0.81-1.19)	0.858	0.88 (0.71 - 1.11)	0.278
**Tearing**				
No	1		1	
Yes	1.03 (0.85-1.26)	0.737	1.09 (0.87 - 1.37)	0.436
**Health facility**				
No	1		1	
Yes	1.15 (0.95-1.40)	0.141	1.13 (0.93 - 1.37)	0.235
**Culture**				
No	1		1	
Yes	1.15 (0.95-1.38)	0.155	1.12 (0.92 - 1.36)	0.269

cPR =Crude Prevalence Ratio, aPR = Adjusted Prevalence Ratio

## Discussion

This cross-sectional study sought to determine the types of Traditional eye medicines, ocular complications, and factors associated with ocular complications among TEM users at Mulago National Referral Hospital Eye Department.

We found that, 21 types of TEM were used by TEM users, of which majority were in liquid forms and few in powdered form. The commonly used TEM was plant products, of which plant liquid products were the most used type of plant products.

These findings are consistent with other studies which found that plant and plant products are the most commonly used TEM products 2, 3, 9. This could be because plant products have been used for medicinal purposes for centuries. Additionally, plants are easy and widely available, and less costly compared to other types of TEM. Human products were the least frequently used TEM in our study. This is because animal products such as breast milk are not readily available as they can only be got from a nursing mother. It is important to note that plant-based products are frequently associated with secondary fungal infection 14.

In our study, we found that most of the participants obtained the TEM from non-traditional practitioners who were mainly relatives, parents, and friends. This is comparable to findings from similar studies in Africa. In a cross-sectional study conducted at the eye unit of Kaguvi Hospital in Zimbabwe, plants and plant products were prescribed by relatives and friends 9. Similar findings were also found among patients using TEM in a tertiary eye hospital in Nigeria 3. This implies that, although the traditional medical practitioner is the originator of TEM therapy, societal input plays a crucial role in the perpetuation of the practice. It also reflects the role of family members as major prescribers of TEM after traditional medical practitioners.

Seven out of 10 participants were found to have ocular complications. This frequency is high, contrary to existing reports from Nigeria showing that complications occurred with frequency of five in every ten TEM users. 2, 3, and another study had a prevalence of 58.6% 9. This high overall prevalence of ocular complications in our study may reflect the actual overall magnitude of ocular complications. It could be also because the study was performed during the Covid-19 epidemic with limited health care service which could have compelled many participants to use TEM. The commonest ocular complication of TEM use was conjunctivitis followed by a corneal ulcer. Presumed chemical conjunctivitis and corneal ulcers found in these patients are potentially sight-threatening complications if not managed appropriately. Majority of study participants reported having used TEMs due to tearing and itching. These findings are similar to existing literature. For instance, it was found that watering and itching were the commonest symptoms that participants in a study about the use of TEM and self-medication presented within rural India 4. While Ukponmwan also noted similar findings among eye patients in Nigeria 2. A study by Mwanza and Kabasele found that TEM worsens already existing ulcers leading to perforations 5.

Factors associated with ocular complications were; residence and availability of TEM. Our data shows that rural dwellers used TEMs more than urban dwellers. This is similar to findings in other studies, for instance, participants from rural settings were more likely to use TEM than urban participants in a study conducted among eye patients at a teaching hospital in Nigeria 2. A study conducted by Gupta in rural India found that access to local healers, poverty, and cultural beliefs are some of the reasons why rural residents use TEM more than urban residents 4. However, in this study participants living in the urban areas were 46% more likely to have ocular complications than those in the rural areas. This could be due to a general increase in the use of traditional medicine (TM) and complementary and alternative medicine (CAM) throughout the world, which in low- and middle-income countries, up to 80% of the population may rely on TM for their primary health care needs and in many high-income countries, CAM utilization is becoming increasingly popular, with up to 65% of the population reporting that they have used this form of medicine 15. Since the study was also hospital-based and done at a national referral hospital with mostly an urban attachment, and most participants may be urban-centered increasing the ratio of urban dwellers closer to that of the rural dweller in the study population, with an increase in the availability of different TEM could have led urban dwellers to be likely to have ocular complications.

Availability of TEM was associated with ocular complications due to TEM among TEM users. In our study, TEM users who had ready access to TEM had a 53% higher likelihood of having ocular complications due to TEM, this is consistent with other literature. A study conducted by Munaw among residents of Gondar city in Ethiopia found that the availability of TEM was significantly associated with TEM use 13. This can be attributed to the wide availability and affordability of TEM while most rural areas have few or no health facilities that offer eye health care. Also, eye care services and eye specialists are only found at the district level hence individuals in rural settings may have no access to these services or may have to travel long distances to such facilities which may demotivate them in seeking care hence resorting to TEM.

Although health facility, education level, cultural beliefs, and occupation were not statistically significant in our study, they are associated with TEM use in similar studies 2, 3, 13. The failure to elicit these associations in this current study may be due to underlying extraneous factors such as social media access. Many testimonies and social marketing of traditional medicines are shared on social media whose use is likely associated with education level and occupation.

## Study strengths / limitations

Our study had some limitations. First, we did not measure ocular complications among subjects who hadn't used TEM for comparison. We also could not determine the eye exam status of the patients before use of the TEM. We therefore cannot entirely relate the ocular complications to TEM use. The main strength of this study was that ocular complications of TEM were ascertained objectively and in real-time by a trained ophthalmologist, making the results accurate and valid.

## Conclusion and recommendations

Plant-based products were the most frequently used types of TEM and were often prescribed by non-traditional healers. A large proportion of the TEM users were found with potentially sight-threatening ocular complications. Urban dwellers[WU7] and ease of availability of TEM were strongly associated with ocular complications.

We, therefore, recommend that the government, through the ministry of health-eye unit, should focus on both urban and rural community education and ophthalmologic clinics outreaches for the target population to reverse the trend[WU8] We should also have a high index of suspicion of TEM use among patients presenting with conjunctivitis in our clinics and manage such patients cautiously and appropriately. Plant products commonly used as TEM should be evaluated further. Lastly, the community and patients attending the ophthalmologic clinic should routinely be educated on the dangers of using TEMs.
